# Carbon Nanotube–Phenyl Modified g-C_3_N_4_: A Visible Light Driven Efficient Charge Transfer System for Photocatalytic Degradation of Rhodamine B

**DOI:** 10.3390/molecules29225439

**Published:** 2024-11-18

**Authors:** Sahar Aghapour Ghourichay, Samira Agbolaghi, Riccardo Corpino, Pier Carlo Ricci

**Affiliations:** 1 Department of Physics, University of Cagliari, S.P. 8 Km 0.700, 09042 Monserrato, CA, Italy; saharaghapour1994@gmail.com (S.A.G.); riccardo.corpino@dsf.unica.it (R.C.); 2 Chemical Engineering Department, Faculty of Engineering, Azarbaijan Shahid Madani University, Tabriz 53714-161, Iran; s.agbolaghi@azaruniv.ac.ir

**Keywords:** f-CNT/PhCN photocatalysts, photogenerated electron-hole pairs

## Abstract

In this study, we report the synthesis and characterization of a novel photocatalyst composite composed of functionalized carbon nanotubes (f-CNT) and phenyl-modified graphitic carbon nitride (PhCN). The incorporation of the phenyl group extends the absorption range into the visible spectrum compared to pure g-C_3_N_4_. Additionally, the formation of the heterostructure in the f-CNT/PhCN composite exhibits improved charge transfer efficiency, facilitating the separation and transfer of photogenerated electron-hole pairs and reducing recombination rates. The photocatalytic performance of this composite was evaluated by the degradation of Rhodamine B (RhB) under visible light irradiation. The f-CNT/PhCN composite exhibits remarkable efficiency in degrading RhB, achieving 60% degradation after 4 h, and 100% after 24 h under low-power white LED excitation. This represents a substantial improvement over the non-functionalized CNT/PhCN composite, which shows much lower performance. In contrast, pure PhCN demonstrates very little activity. Structural and optical properties were characterized using X-ray diffraction (XRD), transmission electron microscopy (TEM), Raman spectroscopy, and UV–Vis spectroscopy. Time-resolved photoluminescence measurements were used to study the behavior of photoexcited carriers, confirming that the composite improves charge transfer efficiency for photogenerated carriers by approximately 30%. The results indicate that the functionalization of CNTs significantly enhances the photocatalytic properties of the composite, making f-CNT/PhCN a promising candidate for environmental remediation applications, particularly in the degradation of organic pollutants in wastewater.

## 1. Introduction

The issue of water pollution—marked by chemical contaminants like dyes, viruses, and heavy metals—is escalating, causing detrimental effects on both the environment and human health [1,2,3]. Photocatalytic technology emerges as a beacon of hope, garnering worldwide attention for its eco-friendly nature and cost-effective capabilities [4]. Enter graphitic carbon nitride (g-C_3_N_4_), hailed for its stability, thermal resilience, chemical inertness, non-toxicity, ease of modification, and remarkable electrical properties [5,6]. However, its standalone efficacy in breaking down organic pollutants remains limited due to the high recombination rates of the photogenerated carriers [7]. To enhance its photocatalytic prowess, diverse strategies have been devised, including template utilization, acid treatment to increase surface area, heteroatom doping to narrow the bandgap, and coupling with other semiconductors to create heterojunction photocatalysts, aiming to suppress the recombination of electron-hole pairs [8,9,10]. Recent studies have demonstrated that modifying the surface of carbon nitride materials, such as heterojunction formation or incorporating carbon-based materials like carbon dots, can significantly enhance photocatalytic activity by improving charge separation and exposing additional active sites [11,12,13].

Zhu et al. devised a novel strategy to enhance the optical and electronic attributes of the g-C_3_N_4_ semiconductor by incorporating sulfur or phosphorus through using substitutional and interstitial doping models [14]. g-C_3_N_4_/ZnO photocatalysts with varied CN loadings were prepared via a simple precipitation–calcination method in the presence of exfoliated C_3_N_4_ nanosheets. The resulting CN/ZnO composite, where ZnO nanoparticles were uniformly distributed on CN nanosheets, showed enhanced photocatalytic activity compared to pure ZnO under visible light [15,16]. Similarly, the LaFeO_3_-coupled graphitic carbon nitride composite exhibited enhanced photocatalytic efficiency in degrading organic pollutants under visible light due to the effective interfacial transfer of photogenerated electrons and holes between LaFeO_3_ and g-C_3_N_4_, which inhibits the recombination [17]. Carbon nanotubes (CNT) have garnered considerable attention as catalyst carriers, thanks to their distinctive one-dimensional structure, expensive specific surface area, robust mechanical attributes, and commendable thermal stability [18,19]. Due to their high current density and substantial specific surface area, these materials can form strong synergies with various semiconductors, including metal sulfides, metal oxides, and other metal substrates. This capability facilitates enhanced performance through collaborative effects, demonstrating their versatility in diverse applications within the realm of materials science and semiconductor technology [20,21,22,23,24,25,26]. In contemporary applications, CNTs have been strategically integrated with g-C_3_N_4_ to amplify the photocatalytic prowess of g-C_3_N_4_. In this regard, a metal-free photocatalyst was created by synthesizing a composite of mesoporous carbon nitride and carbon nanotubes through a simple polymerization method involving melamine as the precursor in the presence of CNT in methanol. The resulting CNT/mpg-C_3_N_4_ composite demonstrated a faster decomposition rate under visible light compared to bulk graphitic carbon nitride and pure mpg-C_3_N_4_. The improved photocatalytic performance is attributed to the synergistic effects of combining open-ended carbon nanotubes with mpg-C_3_N_4_ in the composite [27,28]. Beyond this, CNTs act as distributed templates and carriers, exerting influence over the morphology of semiconductor materials. Furthermore, CNTs serve as photosensitizers, broadening the absorption range of g-C_3_N_4,_ thereby enhancing photocatalytic efficiency [29]. However, CNTs exhibit poor dispersibility and are insoluble in solvents, significantly limiting their applications [30]. Various techniques, including mechanical and chemical methods, involve altering their wetting or adhesion properties, facilitating the charge transfer process and improving their dispersion stability [31,32]. In this regard, Agbolaghi et al. enhanced photovoltaic efficiency through the creation of nanohybrids, utilizing chemically surface-modified CNTs paired with regioregular Poly (3-hexylthiophene) and non-regular Poly (3-dodcylthiophene). These unique donor–acceptor nanohybrids proved effective in the active layer of organic electronics [33]. Nova-Fernandez et al. prepared a hybrid system through direct covalent functionalization of SWNT with 10-phenylphenothiazine via diazonium chemistry. This process resulted in an increase in photocatalytic activity due to the formation of a direct covalent bond between the two units [34].

In the present work, we employed a rapid and straightforward method to prepare a composite photocatalyst by functionalizing carbon nanotubes with polythiophenes and phenyl-modified graphitic carbon nitride. The Catalyst’s morphology, structure, and optical properties were characterized, and RhB photodegradation performance was assessed to showcase the activity of the composite catalyst. This study introduces the successful design and synthesis of a hybrid photocatalyst composed of functionalized carbon nanotubes (f-CNT) and phenyl-modified graphitic carbon nitride (PhCN), significantly enhancing visible-light-driven photocatalytic performance. By improving charge separation and reducing recombination rates, this approach outperforms both pure PhCN and the non-functionalized CNT/PhCN composite, distinguishing this work from previous research in photocatalytic materials. In addition to demonstrating the novel design of the f-CNT/PhCN hybrid photocatalyst, this work focuses on the significant enhancements in photocatalytic efficiency under visible light by using a low-power LED source to avoid UV light exposure. This ensures that the observed degradation results from pure visible-light-induced activity, which makes the system highly relevant for sustainable environmental applications. The study not only highlights the importance of charge separation and the reduction recombination but also rules out potential dye sensitization effects, which could skew photocatalytic performance assessments.

## 2. Results and Discussion

### Characterization of the f-CNT/PhCN Photocatalyst

The analysis of the Raman spectra sheds important indications on the functionalization of the carbon nanotubes and the formation of the hybrid f-CNT/PhCN compound. Two characteristic peaks are typically observed for carbon nanotubes: the D-band at about 1350 cm^−1^ is representative of the sp^3^ disorder carbons, while the G-band is associated with the graphitic sp^2^ carbons. More specifically, the G-band is related to the doubly-degenerate ITO and LO phonon with E2g symmetry at the Brillouin zone center [35,36], while the doubly-resonant second-order D-band involves one ITO phonon and one defect [37]. The strong linear dispersion of the phonon frequency of the D-band (53 cm^−1^/eV) is related to a resonance process between phonons and an electronic transition between linearly dispersive π and π* states. In this case, the Raman spectra are acquired with a near-infrared excitation (1064 nm), and a shift of the D-band to 1300 cm^−1^ is observed for CNT. A further shift to 1260 cm^−1^ with broadening of the Raman band is assigned to the functionalization of the CNT surface, particularly the increased electron doping associated with the thiophene group that enhances π-electron conjugation. A direct observation of the presence of the thiophene is the new band at 1450 cm^−1^ (A-Band) related to collective mode involving the stretching CC modes of the polythiophene backbone [38,39]. The B-band, related to localized vibrational modes, is not clearly visible (at about 1350 cm^−1^), as it partially overlaps with the D-band of the CNT [40].

**Figure 1:**
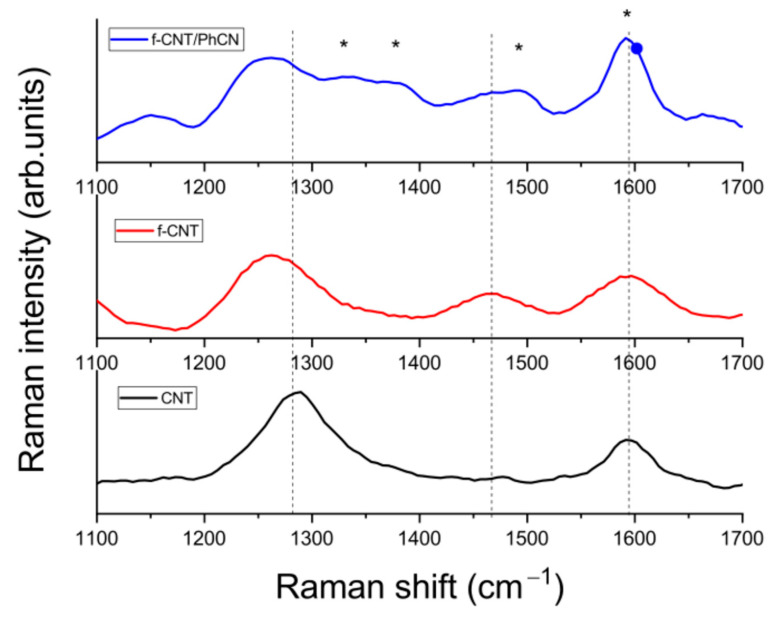
Raman spectra of CNT-COOH, f-CNT, and f-CNT/PhCN. The asterisks (*) indicate the Raman peaks of PhCN.

The Raman spectrum of the hybrid system presents several additional bands, indicated in Figure 1 with asterisks, all of them associated with the PhCN vibrational modes. In particular, the most intense peak at 1600 cm^−1^, generated by the motion of the phenyl group, overlaps with the G-band. Another intense feature is a band at about 1390 cm^−1^, assigned to conjugated stretching vibration (C-C) between the phenyl ring and the triazine rings [41].

Further peaks at about 1350 and cm^−1^ and at 1500 cm^−1^ have been assigned to the bending of CNCN and stretching of NH, respectively [42]. In the hybrid system, we can observe the peak at around 1600 cm^−1^ which can be attributed to the CNT, but by the presence of PhCN, the intensity of the peak has increased.

**Figure 2:**
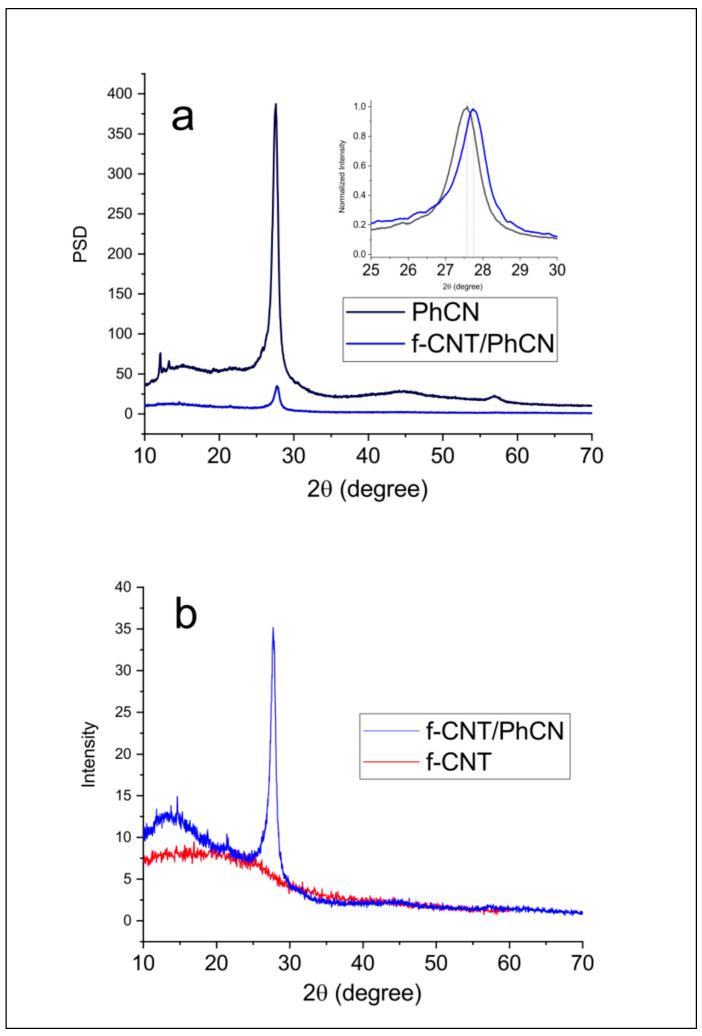
XRD patterns for (**a**) PhCN, f-CNT/PhCN, inset normalized area of PhCN diffraction peak and (**b**) f-CNT, f-CNT/PhCN.

The XRD patterns of as-prepared photocatalysts are shown in Figure 2a,b. The XRD pattern of PhCN reveals two peaks, approximately at 27.5°, corresponding to the (001) plane, and a peak at 13°, associated with the reflection from the (100) plane [43]. In the case of f-CNT, a broad peak is observed at 18°, where we would expect to see a peak around 26°, corresponding to the graphite-like SP^2^ structure of CNT [44]. The diffraction patterns of the f-CNT display characteristics resembling both MWCNT and polythiophenes, marked by a noticeable decrease in peak and shift in peak position [45]. According to the previous studies, the peaks related to the typically multi-walled carbon nanotube are in the range of 26.3, 42.5, and 54.3, but due to the hybridization their intensity is strongly reduced, falling below the observable threshold [46]. The (001) distinctive peak of the PhCN is clearly visible in the XRD of the hybrid system, presenting a shift to 25.7°. Since the peak is related to the distance between planes, a higher diffraction angle indicates a smaller distance between the planes. In pure PhCN, the distance is about 3.26 Å, while in the hybrid f-CNT/PhCN, it is shifted to 3.24 Å. The decrease in the distance can be connected to the role of CNT which acts as a bridge between the PhCN planes, reducing their distance for electrostatic attraction.

**Figure 3:**
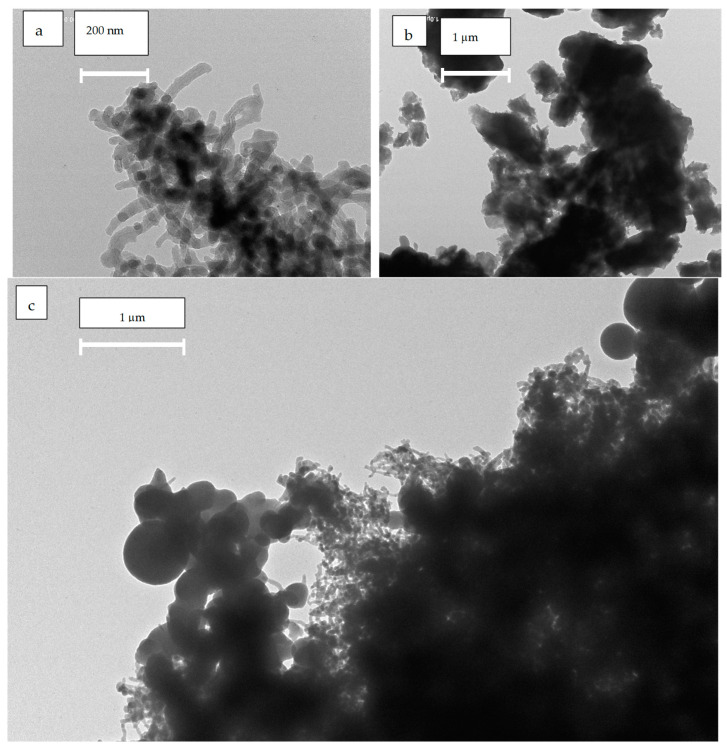
TEM images of (**a**) f-CNT, (**b**) PhCN, and (**c**) f-CNT/PhCN.

The microstructure and morphology of the f-CNT and the hybrid system are illustrated in Figure 3 by TEM images. The f-CNTs have an average diameter of 40 Å with very low dispersion and lengths up to 1 micrometer with a wider distribution, while PhCN agglomerates into micrometer-sized clusters. As depicted in Figure 3, it is evident that f-CNT envelops the PhCN, demonstrating the successful integration of the two materials. The images show a precise overlap of the f-CNTs and the polymer, with no evidence of building block clusters or a spread distribution of the f-CNT component. Due to the composition of the composite materials, functionalized carbon nanotubes (f-CNT) and phenyl-doped graphitic carbon nitride (PhCN), both are predominantly carbon-based—the primary elemental difference is the nitrogen in PhCN. Consequently, elemental analysis yields a high margin of error, particularly in distinguishing between the carbon sources, making precise estimation challenging. The weight percentages provided ensure experimental reproducibility but do not offer a detailed breakdown of the elemental composition in the final composite.

**Figure 4:**
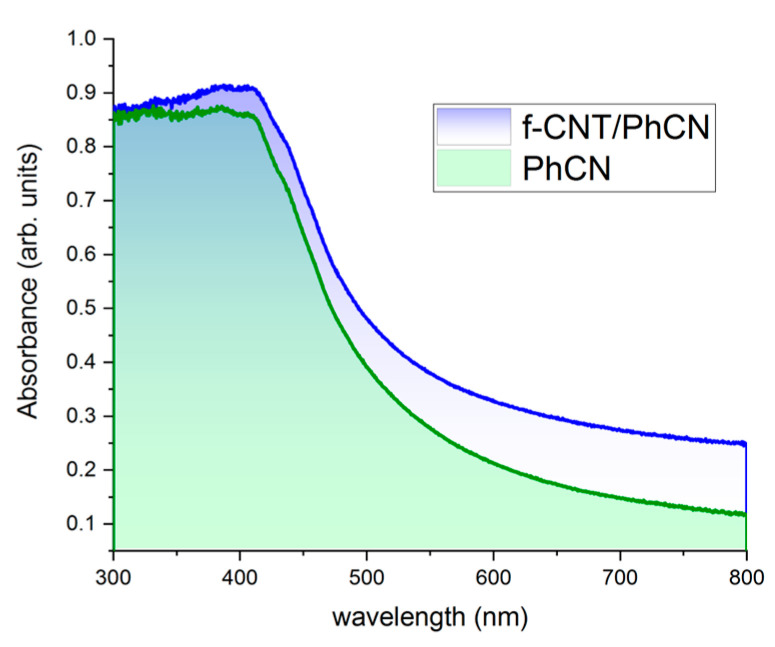
Absorption spectra of PhCN and f-CNT/PhCN.

The UV–Vis spectra were employed to examine the optical characteristics of f-CNT, PhCN, and f-CNT/PhCN hybrid systems. As depicted in Figure 4, the absorption spectra of the composites closely resemble the spectral dependence of pure PhCN; however, f-CNT presents a flat absorption in the visible range and does not contribute to the band gap of polymer (2.70 eV) [47], while increasing the absorbance in the near-infrared region. To understand the interaction mechanism between PhCN and f-CNT upon illumination, the kinetics behavior of the photo-excited carriers has been studied by time-resolved measurements.

**Figure 5:**
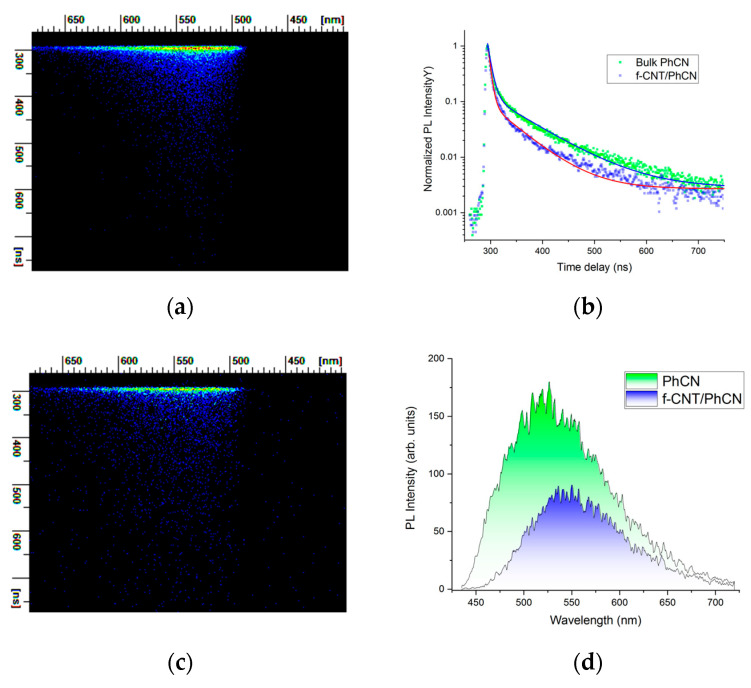
Time-resolved measurement for PhCN and f-CNT/PhCN: ((**a**,**c**) 3D profiles for f-CNT/PhCN and PhCN), (**b**) photoluminescence intensity versus time delay. The blue and red line represent the numerical fit. (**d**) Photoluminescence intensity versus wavelength.

The comparison of decay profiles, acquired using a 410 nm excitation wavelength and recorded over a 500 ns time window (as illustrated in Figure 5), reveals that the f-CNT/PhCN hybrid system exhibits a considerably swifter decay profile in contrast to the decay observed in the PhCN system alone. PhCN in general possesses a slower decay behavior compared to the pure graphitic carbon nitride, with the phenyl group further slowing its kinetic behavior. This slower decay in PhCN is thought to facilitate charge transfer to materials such as metal oxides [48] or the CNT, compared to the hybrid system with g-C_3_N_4_. Figure 5, panels A and C, reports the 3D imaging profile of the recombination kinetics. The horizontal axis reports the spectral dependence, while the vertical axis reports the time delay.

Panel D displays the spectral dependence of photoluminescence for both pure PhCN and the f-CNT/PhCN composite. The luminescence of phenyl-modified graphitic carbon nitride (PhCN) primarily arises from the formation of self-trapped excitons (STEs). Upon photoexcitation, excitons (electron-hole pairs) are generated in PhCN and subsequently localized as STEs due to network distortions. Highly localized STEs emit in the blue spectral region, while less localized excitons result in a red shift. The observed red shift in the f-CNT/PhCN hybrid suggests that the STEs in the composite are less localized than in pure PhCN, likely due to the interaction between f-CNT and PhCN that modify the excitonic behavior. Panel C presents the experimental data and the corresponding fitting procedure, which integrates the entire spectral range (no significant variations were observed in the kinetics as a function of wavelength). The decay time profiles were modeled using a multi-exponential decay function (Equation (1)), capturing the complexity of the recombination dynamics in both the pure and hybrid systems.

(1) $$y=\sum_{i}{A_{i}e}^{-\frac{t}{\tau_{i}}}$$

The experimental time-resolved luminescence data are connected to the probability of recombination from the excited states, by Equation (2):

(2) $$\gamma_{tot}=\gamma_{rad}+\gamma_{NR}=\frac{1}{\tau_{rad}}+\frac{1}{\tau_{NR}}=\frac{1}{\tau_{tot}}$$

where *i* = 1 or 2, *γ_tot_* is the overall recombination probability from the excited state with *γ_rad_* and *γ_NR_* as the probabilities for radiative and non-radiative recombination, respectively. *τ_rad_* and *τ_NR_* are the relative time lifetime of the radiative and non-radiative paths, and *τ*_tot_, the overall decay time, is experimentally found by the fitting procedure. A double exponential curve indicates the presence of two excited levels with different lifetimes. The fast component *τ*_1_ decreases from 6.9 ns to 4.8 ns, while the slower component decreases from 75 ns from the pure PhCN to 54 ns in the f-CNT\PhCN system. This faster decay in the hybrid system suggests a new non-radiative decay channel, associated with the transfer of photogenerated electrons from PhCN to f-CNT. The observed 30% increase in the charge transfer efficiency (*Eff*), calculated as alongside a significant reduction in overall emission intensity, underscores the role of f-CNT as an electron acceptor (Equation (3))

(3) $$Eff=1-\frac{\tau_{f-CNT/PhCN}}{\tau_{PhCN}}$$

Notably, the excitation wavelength falls within the visible range, indicating that the process under study can resume the behavior of the hybrid sample under visible light excitation and confirms the efficiency of the excitation process for wavelengths above 400 nm. The high-rate transfer process (rapid charge transfer), resulting from efficient charge separation and low-energy optical excitation, enhances the f-CNT/PhCN hybrid system’s performance, highlighting its potential as a photocatalyst under visible excitation.

**Figure 6:**
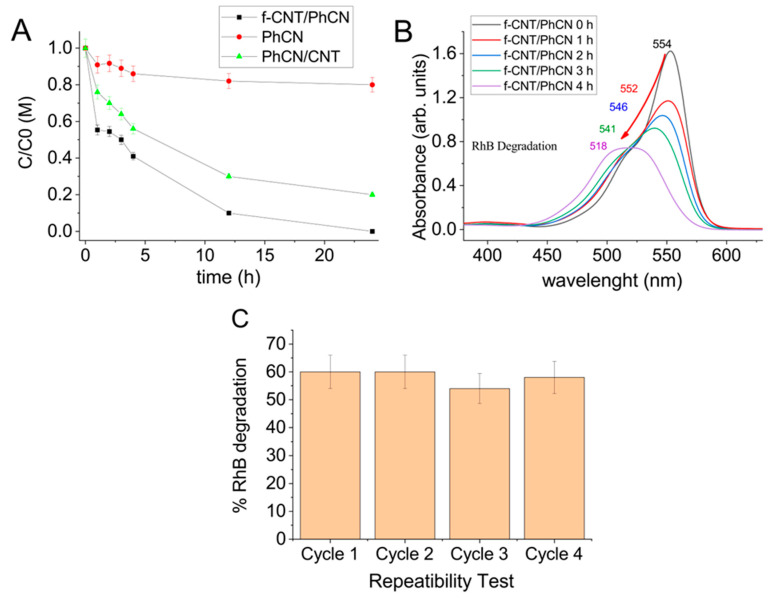
Evaluation of the photocatalytic activity. (**A**) Photocatalytic degradation of pure PhCN, CNT/PhCN and f-CNT/PhCN. (**B**) Absorption spectrum of RhB solution with f-CNT/PhCN. (**C**) Repeatability test of f-CNT/PhCN evaluated after four hours.

The hybrid system was utilized to degrade Rhodamine B in an aqueous solution under visible light (Figure 6). To ensure that no UV components interfere with the process, light from a white LED was used to activate the photocatalysis.

The degradation efficiency of Rhodamine B was determined by monitoring changes in the absorption spectrum over time. Variations in the 554 nm absorption peak shape indicate dye degradation, while reductions in absorption without changes to the spectrum suggest adsorption onto the photocatalyst surface. While the adsorption of RhB onto the catalyst surface is a necessary step in the photocatalytic process, the optical absorption spectra indicate that it constitutes only a minor fraction of the overall degradation. The spectral data show a clear shift in the absorption peak during photocatalysis, indicating molecular breakdown rather than mere adsorption, which would result in a reduction in peak intensity without any spectral shift.

By normalizing the absorbance maximum values to the initial dye concentration and using the Beer–Lambert law, we were able to accurately estimate the concentration of the dye and calculate the degradation efficiency over time (Equation (4)).

(4) $$\mathrm{Degradation}\;\mathrm{Efficiency}=\left(\frac{A_{0}-A_{t}}{A_{0}}\right)\times 100.$$

where A_0_ stands for the initial absorbance and A_t_ for the absorbance measured at different times.

As observed in previous studies, although phenyl-modified graphitic carbon nitride extends absorption above 450 nm, its photocatalytic activity is significantly inhibited by a fast and efficient radiative recombination process. After one day of illumination, the efficiency remains very poor, below 20%, and functionalized CNTs demonstrate no photocatalytic activity under visible light [49]. The marked enhancement in performance is only observed with the f-CNT/PhCN composite, underscoring the synergistic effect between the two components in improving charge separation and reducing recombination rates.

The enhanced effect attributed to the presence of carbon nanotubes (CNT) or, more precisely, the formation of the heterostructure is evident in the high photodegradation efficiency of CNT/PhCN and functionalized CNT/PhCN, underlining that CNTs increase the charge migration and separation, significantly enhancing the photocatalytic performance. The efficiency reaches 40% and 60% after four hours, respectively, and up to 100% after one day of illumination for the functionalized CNT hybrid system. The result is comparable with the similar recent composite based on graphitic Carbon Nitride (Table 1). The functionalization of the carbon surface facilitates the formation of the hybrid system and enhances the stability of the compound, which shows no reduction in performance after four cycles.

Phenyl-modified graphitic carbon nitride (PhCN) is considered an intrinsic semiconductor, similar to g-C_3_N_4_, with its Fermi level positioned approximately in the middle of the bandgap at +0.25 V vs. NHE. Given that the bandgap of PhCN is 2.0 eV, the LUMO (lowest unoccupied molecular orbital) is located at −0.43 V vs. NHE, while the HOMO (highest occupied molecular orbital) is positioned at +1.57 V vs. NHE [63]. The introduction of f-CNT is expected to slightly alter the band edge positions of the nanocomposite, while primarily facilitating electron transport from PhCN. This change is expected to reduce the recombination rate of photogenerated electron-hole pairs.

**Figure 7:**
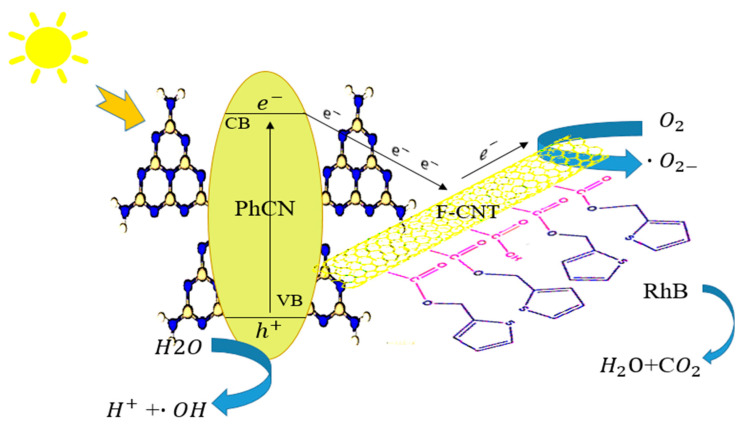
Scheme of the mechanism of f-CNT/PhCN for RhB degradation.

The mechanism of the hybrid system for RhB degradation is illustrated in Figure 7. The carbon atoms in CNTs are primarily derived from sp^2^ hybrid orbitals and, to a lesser extent, from sp^3^ hybrid orbitals. The chemical bonds of carbon atoms in CNTs are a mixture of sp^2^ and sp^3^ hybridizations. These *p*-orbitals overlap to form graphene-like external surfaces of CNTs, resulting in highly delocalized large π bonds. These large π bonds can form conjugated macromolecular composites through noncovalent interactions. Since phenyl-doped carbon nitride has a π-bond-rich conjugated structure, it naturally tends to interact noncovalently with CNTs to form composites.

As is well known, the defects in organic polymers cause free electrons to transfer very slowly on the surfaces and within the volume of carbon nitride. The photocatalytic ability of PhCN is limited by rapid electron-hole recombination and low electron utilization rate. Further, the presence of self-trapped excitons (STE) affects photocatalytic efficiency. STEs are characterized by the localization of electron-hole pairs within the material that reduces the mobility of the charge carriers. As a result, photogenerated electrons and holes are less likely to migrate to the surface of the photocatalyst where they can participate in redox reactions. This diminished mobility can lead to lower photocatalytic efficiency; therefore, fewer charge carriers are available to drive the photocatalytic reactions. The non-covalent interaction causes CNTs to tightly adsorb onto the polymer, forming groups that accelerate electron migration on the surface and within the volume of PhCN, decreasing the effect of STE [64,65,66,67].

Scavenger tests were performed using three different solutions: isopropanol for hydroxyl radicals (•OH), benzoquinone (BQ) for superoxide radicals (O_2_•^−^), and ascorbic acid for photogenerated holes (h^+^). The measurements, conducted over 4 h, revealed distinct reductions in photocatalytic efficiency. The presence of ascorbic acid resulted in a moderate decrease in efficiency (approximately 30%), further dropping to 12% with BQ, and becoming nearly null in the presence of isopropanol. These results indicate that hydroxyl radicals (•OH) are the primary species responsible for oxidative degradation in the photocatalytic system, though superoxide radicals also play a significant role.

As evidenced by the scavenger tests, under light irradiation, the photocatalyst absorbs photons that excite electrons (e^−^) from the valence band (VB) to the conduction band (CB) of PhCN, leaving behind holes (h^+^) in the VB. The recombination of these charge carriers is greatly reduced due to the electron transfer process to the f-CNTs (as shown by time-resolved measurements), allowing the positive charge (holes) to remain available for oxidation reactions. The holes (h^+^) in the VB are highly oxidative and can react with surface-bound water molecules (H_2_O) or hydroxide ions (OH^−^), generating hydroxyl radicals (•OH):

$$h^{+}+H_{2}O\to ∙OH+H^{+}\;h^{+}+OH^{-}\to ∙OH$$

The hydroxyl radicals (•OH) are responsible for attacking and oxidizing the organic pollutant (e.g., Rhodamine B) during the photocatalytic process. In addition to generating hydroxyl radicals, the electrons (e^−^) transferred to the f-CNTs can react with dissolved oxygen (O_2_) in the solution, forming superoxide radicals (O_2_•^−^). The synergistic effect of f-CNTs and PhCN significantly enhances degradation efficiency, as demonstrated by the complete photodegradation of RhB after one day of illumination under low-power-density visible light.

In summary, the degradation mechanism of Rhodamine B in the f-CNT/PhCN photocatalytic system is driven by the generation of electron-hole pairs upon visible light irradiation (Figure 7). The phenyl-modified graphitic carbon nitride (PhCN) acts as the primary photocatalyst, transferring photoexcited electrons to the functionalized carbon nanotubes (f-CNTs), improving charge separation and minimizing recombination. The electrons in the f-CNTs facilitate the reduction of RhB, while the holes in PhCN promote oxidative processes, leading to the effective degradation of the dye molecules.

## 3. Experimental Section

### 3.1. Materials and Methods

#### 3.1.1. Materials

Ph-Triazine (99%), Rhodamine B (95%), Carbon Nanotube Multi-walled, and L-ascorbic acid (99%) were procured from Merck/Sigma Aldrich (Darmstadt, Germany), with absolute methanol (94–96%) sourced from Alfa Aesar (Haverhill, MA, USA). The preparation of the Rhodamine Aqueous solution utilized deionized water. Sulfuric acid (95–97%), nitric acid (65%), and anhydrous dimethyl sulfoxide were acquired from Merck/Sigma Aldrich (Darmstadt, Germany). Additionally, 2-hydroxymethyl thiophene and *p*-toluensulfonic acid (*p*-TSA) were purchased from Merck/Sigma Aldrich.

#### 3.1.2. Preparation of Phenyl-Modified Carbon Nitride (PhCN)

The fabrication of phenyl-modified carbon nitride involved meticulously placing 1 g of 6-phenyl-1,3,5-triazine-2,4-diamine powder within a quartz tube, carefully positioned in a tubular furnace. The synthesis process commenced by subjecting the arrangement to a controlled heating regime, gradually elevating the temperature to 400 degrees Celsius over 1 h. This procedure facilitated the development of the desired material with improved phenyl modification [63].

#### 3.1.3. Preparation of Thiophene Functionalized Carbon Nanotubes (f-CNT)

The functionalization process of multi-walled carbon nanotubes involved an oxidation method accompanied by sonication with sulfuric acid and nitric acid. After undergoing five cycles of stirring and decantation, the resultant precipitate underwent drying in a vacuum oven maintained at 60 degrees Celsius. Following this step, the synthesis of the 2-hydroxymethyl thiophene macroinitiator commenced through the esterification of CNT-COOH with 2-hydroxymethyl thiophene. *p*-toluensulfonic acid was employed as a dehydrating agent. In a reaction flask, a well-balanced combination of CNT-COOH (0.5 g), 2-hydroxymethyl thiophene (1 g), and anhydrous dimethyl sulfoxide (50 mL) underwent sonication for 40 min, resulting in a homogenous suspension. The introduction of a catalytic quantity of *p*-TSA into the flask was followed by placing it in a silicon oil bath at 140 degrees Celsius, where the reaction mixture was stirred for 6 h under an argon atmosphere. The suspension underwent centrifugation and subsequent washes with methanol to thoroughly remove any residual 2-hydroxymethyl thiophene. The final powder was obtained by drying under reduced pressure at 60 degrees Celsius [33].

#### 3.1.4. Preparation of f-CNT/PhCN Nanocomposite

We incorporated 0.066 g of PhCN into 20 mL of methanol and subjected it to sonication for 30 min. Concurrently, 0.002 g of f-CNT was introduced into 20 mL of methanol and sonicated for the same duration. Subsequently, the two solutions were combined drop by drop while continuous stirring. The resulting mixture was stirred for nearly 2 h, transferred into a 250 mL flask, and heated at 70 degrees Celsius in a water bath for 5 h. The final step involved drying the samples in an oven set at 60 degrees Celsius.

### 3.2. Structural Characterization

X-ray diffraction patterns were operated at room temperature using a Rigaku Miniflex II diffractometer with *θ*–2*θ* Bragg–Brentano geometry with Cu Kα (λ=1.54118 Å), radiation at room temperature.

Raman Spectra were recorded using a Sol Instruments, MS750 series Monochromator-spectrograph (Sol-Instrument, Augsburg, Germany), excitation wavelengths at 785 nm with a resolution of about 1 cm^−1^. Time-resolved photoluminescence measurements were captured by stimulating the samples using 200 fs pulses generated from an optical parametric amplifier (light conversion TOPAS-C, Coherent Saxonburg, PA, USA), which was pumped by a regenerative Ti: Sapphire amplifier (Libra-HE Coherent). The repetition frequency was set at 1 kHz, and the signal was registered using a streak camera (C10910, Hamamatsu, Hizuoka Prefecture, Japan) equipped with a grating spectrometer (Acton Spectra Pro SP-2300, Princeton instrument, Trenton, NJ, USA). To mitigate inner filter effects, all measurements were conducted in the front-face configuration. Additionally, appropriate emission filters were employed to eliminate the reflected contribution of the excitation light.

Transmission electron microscopy (TEM) images were recorded on a Hitachi H-7000 equipped with a thermionic tungsten filament running at 100 kV. Images were collected by a CCD camera (2048 × 2048 pixel) AMT DVC (Woburn, MA, USA). Absorption measurements were acquired through diffuse reflectance spectroscopy employing UV–Vis-NIR JASCO FP-8550ST (Jasco, Easton, MD, USA). The measurements were executed utilizing a PbS solid-state photodetector. In reflection configuration, the diffuse reflection of the sample was assessed in comparison to a BaSO_4_ reference. The application of the Kubelka–Munk equation facilitated the extraction of absorption features.

### 3.3. Photodegradation of RhB

The evaluation of photocatalytic performance entailed monitoring variations in the concentration of RhB within an aqueous solution under visible light irradiation. A white LED, specifically a Philips 13 W light source with an optical power of 100 mW, was employed for this assessment. To establish absorption–desorption equilibrium between the catalyst and the dye, both the catalyst suspension (4 mg) and RhB solution (20 mL of a 10 mg/L solution) underwent a 30 min stirring process in complete darkness before light exposure. Throughout the photodegradation process, 1.5 mL portions were systematically collected at regular 60 min intervals. Subsequently, these portions underwent centrifugation, and the residual concentrations of RhB were analyzed using the advanced UV–Vis Technologies Cary 5000 Spectrophotometer (Agilent, Santa Clara, CA, USA). The concentration of RhB before and after the reaction was investigated by the UV–Vis spectrophotometer at the wavelength of 554 nm. The degradation kinetics of the catalyst were obtained by the first-order kinetics Equation (5):

(5) $$-ln(c_{t}/c_{0})=kt.\;$$

where $c_{0}\;$(mg L^−1^) was the initial concentration of RhB, $c_{t}$ (mg L^−1^) was the concentration at time *t* (min), and *k* was the rate constant (min^−1^).

For the scavenger test, a 1 mM concentration of each scavenger was prepared. We mixed 20 mL of the scavenger solution with 20 mL of Rhodamine B solution. Isopropanol was used as the scavenger for hydroxyl radicals, while benzoquinone and ascorbic acid were used for superoxide radicals and holes, respectively. Subsequently, 4 mg of the nanocomposite was added to the mixture. The solutions were stirred in the dark for 30 min before being exposed to visible light for photocatalytic testing.

## 4. Conclusions

In this paper, the successful synthesis of f-CNT/PhCN nanocomposites using a simple water-bath method is reported, achieving significant enhancement in photocatalytic activity for Rhodamine B degradation. The integration of functionalized carbon nanotubes at an optimal content of 3% with phenyl-modified graphitic carbon nitride resulted in a marked improvement in visible light absorption and efficient photogenerated electron-hole separation. This synergistic effect contributed to the superior photocatalytic performance of the nanocomposites, highlighting their potential as efficient catalysts for environmental applications, particularly in wastewater purification. Despite the use of a low-power LED source, the photocatalyst exhibited remarkable activity, with the functionalization of CNTs playing a fundamental role in enhancing electron transport and reducing recombination rates. This demonstrated improvement, even without UV light, reinforces the applicability of this system for sustainable photocatalytic applications. These results highlight the innovative combination of phenyl-modified g-C_3_N_4_ and thiophene-functionalized CNTs, offering a promising direction for future enhanced sustainable environmental remediation technologies. This study opens new avenues for the development of advanced photocatalytic materials with enhanced performance, underscoring the importance of functionalization and composite formation in optimizing photocatalytic efficiencies.

Future work will focus on exploring the scalability of this synthesis method and the applicability of these nanocomposites to other organic pollutants, aiming to further contribute to sustainable environmental remediation technologies.

## Figures and Tables

**Table 1:** Comparison of the results of the present work with some recent literature.

	Materials	Synthesis Method Used	Experimental Conditions	Light Source	Type of Dye	Degradation Time	Photodegradation	Reference
1	g-C_3_N_4_/Fe_3_O_4_/rGO	Precipitation method	Different ratios of photocatalysts dispersed in RhB solution	Visible light	RhB	75 min	100%	[50]
2	SnO_2_@g-C_3_N_4_	Solid-state reaction	Concentration of dye: 10^−5^ M 0.2 of nanocomposite concentration: 10, 20, 30%	Visible light	Methyl orange	6 h	85%	[51]
3	g-C_3_N_4_/Na_2_Ti_3_O_7_/V_2_O_5_	Hydrothermal method	photocatalysts (0.05 g) dispersed in an aqueous solution (50 mL) containing 0.25 mmol of Na_2_SO_3_ and Na_2_S	Visible light	RhB	60 min	90%	[52]
4	CNT/mp-g-C_3_N_4_	Refluxing/thermal polymerization	0.03 g of sample powder was added to the solution of RhB (50 mL)	Visible light	RhB	5 h	90%	[27]
5	g-C_3_N_4_/TiO_2_	Hydrothermal method	10 mg powder of photocatalyst dispersed in 60 mL dye aqueous solution concentration of (5 ppm)	Visible light	Methylene Blue	2 h	100%	[53]
6	s-PANI@g-C_3_N_4_	In-situ oxidative polymerization	0.2 g of nanocomposite added into the 300 mL aqueous solution that comprised 50 ppm phenol	UV	Phenol	7 h	100% by addition of 1%w GN	[54]
7	g-C3N4/CNT/Bi_2_WO_6_	Hydrothermal method	40 mg photocatalyst added into 40 mL of TC solution	Visible light	TC aqueous solution	90 min	87.65%	[55]
8	2D/1D g-C_3_N_4_/TNT	Hydrothermal method	SMT concentration was 5 mg/L and material dosage was 0.2 g/L, PH = 7	Solar light	SMT	5 h	100%	[56]
9	Activated carbon/g-C_3_N_4_	By mixing melamine and AC	ATZ = 5 mg/L pH = 5.56 Catalyst = 1 g/L	Visible light	Atrazine with PMS	2 h	78.76%	[57]
10	Ag/g-C_3_N_4_/t-CFP	Thermal polymerization with IEP method	As-prepared sample was hung in 20 mL mixed solution containing 20 mg/L MB	Visible light	Methylene Blue	40 min	72%	[58]
11	PPy/P-C_3_N_4_/rGO	In-situ chemical polymerization	50 mg of sample dispersed in 125 mg/L of X3B solution	Visible light	X3B (Brilliant red dye)	1 h	98%	[59]
12	Ag/graphite carbon nitride	Thermal exfoliation and photo-reduction method	50 mg of photocatalyst dispersed in 20 mg/L of TC solution	Visible light	Tetracycline (TC)	2 h	83%	[60]
13	CoFe_2_O_4_/g-C_3_N_4_	Sol-gel and Ultrasonic treatment	30 mg of catalyst dropped into 50 mL of RhB	Visible light	RhB	2 h	57%	[61]
14	ZnO-Bi_2_O_3_/g-C_3_N_4_	Hydrothermal method	50 mg of catalyst added in a 100 mL of Indigo carmine solution (50 mg/L)	Visible light	Indigo Carmine	3 h	68.8%	[62]
15	f-CNT/PhCN	Water-bath method	4 mg of sample added into 20 mL of RhB solution	Visible light	RhB	4 h	60%	This work

## Data Availability

Raw data are available upon request.
